# Comprehensive bioinformatics analysis of integrator complex subunits: expression patterns, immune infiltration, and prognostic signature, validated through experimental approaches in hepatocellular carcinoma

**DOI:** 10.1007/s12672-024-01118-6

**Published:** 2024-06-26

**Authors:** Yifei Xu, Wenlian Liao, Ting Wang, Liwei Zhang, Hui Zhang

**Affiliations:** 1 Institute of Interdisciplinary Integrative Medicine Research, Shanghai University of Traditional Chinese Medicine, Shanghai, 201203 China; 2 Department of Anesthesiology, School of Medicine, Xiang’an Hospital of Xiamen University, Xiamen University, Xiamen, 361101 Fujian China

**Keywords:** INTSs, HCC, Diagnosis, Prognosis, Immune infiltration, Bioinformatics analysis

## Abstract

**Background:**

Hepatocellular carcinoma (HCC) is a common gastrointestinal malignancy with a high incidence and poor prognosis. The subunits of the integrator complex (INTS1-14) play a crucial role in regulating genes dependent on RNA Polymerase II, which may be associated with cancer. However, the role of INTSs in HCC remains unclear. This study aims to comprehensively analyze the clinical value and potential role of INTS family genes in HCC through systematic bioinformatics analysis.

**Methods:**

We employed various public databases, including UALCAN, HPA, Kaplan–Meier Plotter, GEPIA2, TNMplot, STRING, TIMER, and TISIDB, to investigate the expression levels, clinicopathological correlations, diagnostic and prognostic value, genetic alterations, co-expression network, molecular targets, and immune infiltration of INTSs in HCC. Additionally, Gene Ontology (GO) and Kyoto Encyclopedia of Genes and Genomes (KEGG) were utilized to investigate the biological functions of genes associated with INTSs. Furthermore, Western blot, real-time fluorescence quantitative reverse transcription polymerase chain reaction (RT-qPCR), and immunohistochemistry techniques were employed to assess the expression of relevant proteins and genes. The proliferation of HCC cells was evaluated using the CCK8 assay.

**Results:**

We found that in HCC, there was a significant upregulation of INTSs at the transcriptional level, particularly INTS1, INTS4, INTS7, and INTS8. Additionally, the protein levels of INTS1 and INTS8 were notably elevated. The overexpression of these INTSs was strongly correlated with tumor stages in HCC patients. INTS1, INTS4, INTS7, and INTS8 exhibited significant diagnostic and prognostic value in HCC. Moreover, their expression was associated with immune infiltrations and activated status, including B cells, CD8 + T cells, CD4 + T cells, NK cells, macrophages, and dendritic cells. Functional predictions indicated that INTS1, INTS4, INTS7, and INTS8 were involved in various cancer-related signaling pathways, such as TRAIL, IFN-gamma, mTOR, CDC42, Apoptosis, and the p53 pathway. Furthermore, we observed a significant upregulation of INTS1, INTS4, INTS7, and INTS8 expression in HCC cell lines compared to normal liver cell lines. The level of INTS1 protein was higher in cancerous tissues compared to adjacent non-cancerous tissues (n = 16), and the suppression of INTS1 resulted in a significant decrease in the proliferation of Huh7 cells.

**Conclusion:**

These findings indicate the potential of INTS family genes as diagnostic biomarkers and therapeutic targets in HCC. Further research is needed to understand the underlying mechanisms and explore clinical applications.

**Supplementary Information:**

The online version contains supplementary material available at 10.1007/s12672-024-01118-6.

## Introduction

Hepatocellular carcinoma (HCC) is a primary liver cancer and is widely recognized as one of the most common gastrointestinal malignancies worldwide, with a poor prognosis and high incidence [1]. It ranks as the fourth leading cause of cancer-related deaths, accounting for 9.8% of all cancer deaths [2]. HCC progression is also deeply connected with the viral infection [3–6]. Early diagnosis and effective treatment strategies are crucial for improving patient outcomes, underscoring the necessity for new markers [7–9], clinical indicators, and effective therapeutic targets in HCC treatment research.

In recent years, the integrator complex, composed of multiple subunits, has recently emerged as a potential key player in various biological processes such as gene regulation, RNA processing, and chromatin remodeling [10]. Dysregulation of integrator complex subunits (INTSs) has been implicated in several cancers [11, 12]. However, the expression patterns, prognostic significance, and association with immune infiltration in HCC regarding INTSs remain largely unexplored.

To investigate the potential roles of INTS family genes in HCC pathogenesis and their implications for patient prognosis, this study comprehensively examines the expression levels, mutations, diagnostic significance, and prognostic value of INTS family genes in HCC patients. Bioinformatics analyses are conducted using multiple public databases, including UALCAN, HPA, Kaplan–Meier Plotter, GEPIA2, TNMplot, cBioPortal, and TIMER. Furthermore, the study delves into the potential biological functions and gene regulatory networks linked to INTS family genes in HCC, and analyzes their correlation with infiltrating immune cells in the tumor microenvironment. The impact of INTS family gene knockdown on HCC cell proliferation is evaluated using the DepMap database and siRNA. In addition, qRT-PCR and immunohistochemistry are used to detect the mRNA and protein expression of INTS family genes.

The ultimate goal of this study is to provide a comprehensive understanding of the potential roles of INTS family genes in HCC pathogenesis and their implications for patient prognosis. By exploring their biological functions, gene regulatory networks, and correlation with immune infiltration in the tumor microenvironment, we aim to identify novel therapeutic targets for all stages of HCC treatment. Through a combination of bioinformatics analysis and experimental validation, we seek to elucidate the significance of INTS family genes in HCC and pave the way for targeted therapies that may improve patient outcomes in the future.

## Materials and methods

### cBioPortal

We utilized the cBioPortal (https://www.cbioportal.org/), a comprehensive network resource for exploring multidimensional cancer genomics data [13], on December 6, 2023. The INTS family's genetic alterations in HCC cell lines were analyzed using this platform. The z-score of mRNA expression (RNA Seq V2 RSEM) was obtained using ± 1.8 z-score. Furthermore, we analyzed alterations in INTS family genes and their correlation with overall survival (OS) and disease-free survival (DFS) in HCC patients using Kaplan–Meier curves. A statistical significance level of *P* < 0.05 was set.

### UALCAN

The UALCAN platform (http://ualcan.path.uab.edu) allows users to examine the relative expression levels of genes or gene sets in specific tumor subpopulations. These predefined tumor subpopulations include cancer stage, tumor grade, race, and other clinicopathological features [13]. We used the UALCAN database to detect the differential expression of INTS family members in normal and liver tumor tissues, on December 6, 2023.

### TIMER

The TIMER tool (http://timer.comp-genomics.org/) is a comprehensive resource based on the TCGA database, which includes 371 HCC samples and 50 normal liver samples. It provides multiple functions, including comparing gene expression between tumor and normal tissues in different cancers, analyzing the correlation between genes and immune infiltrating cells, conducting survival analysis, and other functionalities [14]. We utilized the TIMER database's "Gene module" to examine the correlation between INTS family members and levels of immune cell infiltration in HCC, including cancer-associated fibroblast, myeloid dendritic cell, CD4 + T cell, neutrophil, T cell regulatory (Tregs), CD8 + T cell, and macrophage, on December 6, 2023.

### TISIDB

TISIDB (http://cis.hku.hk/TISIDB/) is a public portal that integrates tumor and immune system interaction data from numerous heterogeneous sources [15]. In this study, we performed a detailed analysis of immune infiltration of INTSs in HCC based on high-throughput data using the TISIDB database on December 6, 2023.

### Kaplan–Meier plotter

To analyze the prognostic value of INTSs in HCC patients, we used the Kaplan–Meier plotter tool (https://kmplot.com) [16] on December 6, 2023. HCC patients were divided into two groups, and the data were evaluated using a Kaplan–Meier survival plot. Statistical significance was considered for *P* < 0.05.

### GEPIA2

GEPIA (http://gepia2.cancer-pku.cn/#index) is a web server that analyzes RNA sequencing data from the GTEx and TCGA databases, allowing users to analyze human cancer gene expression and interaction [17]. We used GEPIA2 to compare the expression of INTSs in HCC on December 6, 2023.

### Human protein atlas (HPA)

The Human Protein Atlas (https://www.proteinatlas.org/) is a database that provides protein expression profiles in different cancers, normal tissues, and cell lines based on immunohistochemistry (IHC) [18]. In this study, we utilized the HPA database to directly compare the protein expression of different INTS family members in human normal and HCC tissues on December 6, 2023.

### Co-expression analysis

To conduct co-expression analysis of INTSs, we utilized the Linkedomics (http://linkedomics.org/login.php) [19] and cBioPortal databases [20]. Through the LinkedOmics and cBioPortal databases, we identified the top 200 genes that were positively co-expressed and identified the intersecting genes. To understand the functions and pathways associated with the four-INTSs, we performed GO and KEGG enrichment analyses. The enrichment analyses for the genes co-expressed with INTS members were carried out using WebGestalt (http://www.webgestalt.org/#) [21], on December 6, 2023.

### DepMap

The DepMap (https://depmap.org) is a publicly-available CRISPR/Cas9 dropout screen dataset [22]. By analyzing the DepMap database, we identified that knocking out INTSs using CRISPR/Cas9 significantly suppressed the growth of HCC cell lines on December 6, 2023.

### TNMplot

TNMplot (https://tnmplot.com/) is an online database that contains gene expression data of normal, tumor, and metastatic tissues. It is used to compare gene expression in these specific groups and search for the co-expression of targeted genes [23], on December 6, 2023. TNMplot was utilized to compare the expression of INTSs in normal, tumor, and metastatic HCC.

### UCSC Xena

The fragments per kilobase of transcript per million mapped reads (FPKM-UQ) for the upper quartile, in conjunction with relevant prognostic data for liver hepatocellular carcinoma (LIHC), were sourced from the UCSC Xena browser (https://xenabrowser.net/datLIHCapages/). This involved utilizing cancer-specific data from The Cancer Genome Atlas cohort, accessible from the Genomic Data Commons (GDC-TCGA) (Goldman et al., 2020).

### GTEx

Additionally, the normal cohort, covering a comprehensive set of samples, was retrieved from the Genotype-Tissue Expression Project (GTEx) (https://www.gtexportal.org/). We utilized the GTEx database to access extensive RNA-seq data and relevant clinical information. Following this, the RNA-seq data was converted from Fragments Per Kilobase per Million (FPKM) format to Transcripts Per Kilobase per Million (TPM) format [26]. Subsequently, we conducted a thorough analysis of the expression patterns for HCC, on December 6, 2023.

### FunRich

FunRich (http://funrich.org/index.html) is a stand-alone software tool primarily used for functional enrichment and interaction network analysis of genes and proteins. The results of the analysis can be visually depicted in various chart formats, including Venn, bar, column, pie, and doughnut charts. FunRich can handle a variety of gene/protein datasets for different organisms. Users can search against the default background database or load a customized database for functional enrichment analysis [24, 25].

### Cell culture

Human liver cancer cell lines (SMMC-7721, HUH7, JHH7, HepG2, and PLC/PRF/5) purchased from the Chinese Academy of Science Cell Bank (Shanghai, China), were cultured in Dulbecco's modified Eagle's medium (DMEM, Gibco, USA) supplemented with 10% fetal bovine serum (Gibco, USA) and 1% penicillin–streptomycin (Gibco, USA). The cells were incubated in a humidified incubator at 37 °C with 5% CO_2_.

### RNA Extraction and qRT-PCR

Total RNA was extracted from cells using Trizol reagent (Invitrogen, United States), and the concentration and quality of the RNA were assessed using a NanoDrop™ 1000 Spectrophotometer (Thermo Fisher Scientific, USA). The OD260/OD280 ratios were maintained within the range of 1.8–2.0, and the OD260/OD230 ratios were maintained within the range of 2.0–2.2. First-strand complementary DNAs (cDNAs) were synthesized through reverse transcription using 1 μg of RNA and a cDNA reverse transcription kit (Applied Biosystems, Foster City, CA, USA). Quantitative reverse transcription-polymerase chain reaction (qRT-PCR) was conducted using SYBR® Green PCR Master Mix (Applied Biosystems, Foster City, CA, USA) and a StepOnePlus Real-Time PCR System (Applied Biosystems, Foster City, CA, USA). The levels of mRNAs were calculated using the formula 2^ΔCt^, where ΔCt = Ct of internal reference—Ct of target mRNA. GAPDH mRNA was used as a target mRNA internal control. Data obtained by real-time PCR were translated in log 2 (relative level). The primer sequences are summarized in Supplementary Table S1.

### Transfection of siRNA

Human INTS1 siRNA and a scramble control siRNA were synthesized and purchased from GenePharma (Shanghai, China). The sequences for the siRNA and the scrambled negative control were as follows: INTS1 siRNA, 5′-CAUUUCUCCGUCGAUUAAATT-3′ (Sense), 5′-TTTAATCGACGGAGAAATGTT-3′ (Anti-sense); scramble control siRNA, 5′-UUCUCCGAACGUGUCACGUTT-3′ (Sense), 5′-ACGUGACACGUUCGGAGAATT-3′ (Anti-sense). The siRNA was transfected into cultured Huh7 cells at a concentration of 100 nM using Lipofectamine 3000. After 72 h, the efficiency of the siRNA transfection was evaluated by Western blot analysis.

### Western blot

A Total Protein Extraction Kit (Beyotime Biotechnology, Shanghai, China) was used to extract protein from collected cells, which included PMSF, protease inhibitors, and phosphatase inhibitors. The extracted protein was then mixed with 5 × SDS-PAGE and boiled for 5 min. Standard methods for Western blot analysis were applied to assess the expression of INTS1, with β-actin protein serving as an endogenous reference.

### CCK8 assay

Huh7 cell suspensions containing 1 × 10^4^ cells were plated in each well of a 96-well plate and cultured overnight. Following transfection with INTS1 siRNA, cell proliferation was assessed at 24, 48, 72, and 96 h using the CCK8 assay. At each time point, 10 μl of CCK8 solution was added to each well and incubated in a 37 °C incubator with 5% CO_2_ for 2 h. The absorbance of the cells was then measured at a wavelength of 450 nm using a microplate reader. Cell proliferation was expressed as the percentage of treated cells compared to control cells, normalized accordingly.

### Patients and tissue specimens

Primary HCC samples and paired normal tissues were obtained from 16 HCC patients who underwent surgical resection at Shuguang Hospital, Shanghai University of Traditional Chinese Medicine from 2016 to 2019. All research involving human participants have been approved by the Ethics Committee of Shuguang Hospital, Shanghai University of Traditional Chinese Medicine, and all clinical investigations have been conducted according to the principles expressed in the Declaration of Helsinki. (Approve No. 2016-482-33-01) All patients provided “written informed consent” to participate in this study. All samples were fixed in 4% formaldehyde and embedded in paraffin wax. Immunohistochemistry (IHC) was used to study the expression of INTS1 in paraffin-embedded clinical samples. IHC was performed as previously described [27, 28]. Specific primary antibody against INTS1 was purchased from Abcam (cat#ab121692).

### Statistical analysis

The predictive performance of the signature was evaluated using ROC curves, employing GraphPad Prism 8.0 software (GraphPad Software Inc., San Diego, CA, USA). Univariate and multivariate analyses were conducted to identify an independent predictor for the prognosis of hepatocellular carcinoma (HCC) patients. Subsequently, a nomogram prediction model was established, incorporating both clinicopathological characteristics and the risk score. To comprehensively assess and validate the prognostic signature, external validation was performed. Statistical analysis was also performed using GraphPad Prism 8.0 software. Mean values between the two groups were compared using a two-tailed t-test for normally distributed data or the Mann–Whitney U test for non-normally distributed data. Results were presented as mean ± SD, and a significance level of *P* < 0.05 was considered statistically significant.

## Results

### Aberrant expression and promoter methylation status of the INTS family members in HCC

**Fig. 1:**
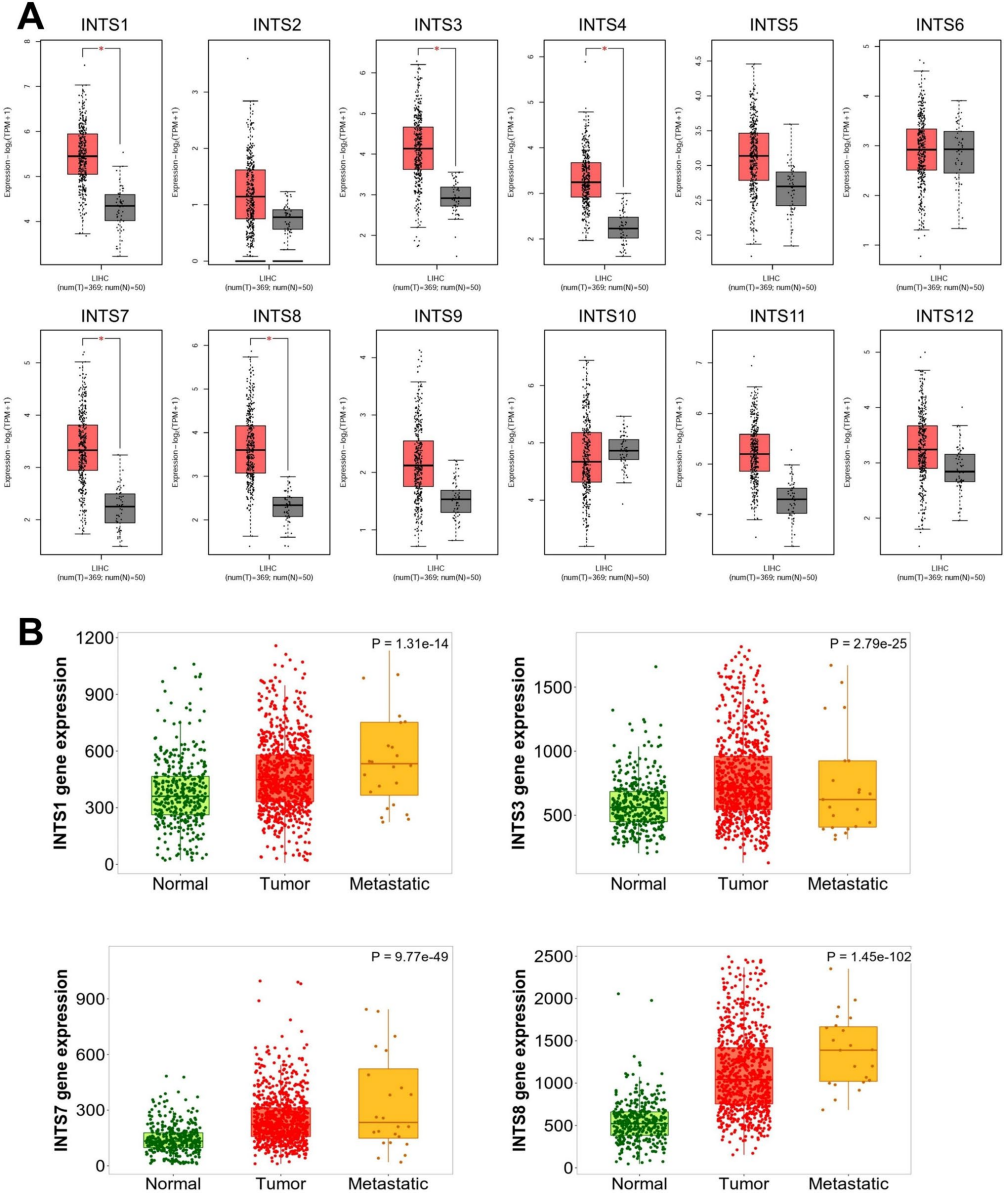
Aberrant expression of the INTS family members in HCC. **A** The mRNA expression of INTS family (INTS1-12) that are aberrantly expressed in HCC tissues (n = 369) versus normal liver tissues (n = 50) using GEPIA database. *P < 0.05. **B** The transcript expression levels of INTS1, INTS3, INTS7, and INTS8 were analyzed in tumors and metastatic tissues compared to normal tissues using the TNM plotter database. The study included a total of 379 normal samples, 806 tumor samples, and 24 metastatic samples

To investigate the expression of INTS family members in HCC, we used multiple databases to analyze the mRNA expression of INTS family (INTS1-14) in HCC. We first analyzed the relative mRNA expression levels of INTSs between normal and tumor tissue using the UALCAN system. The results showed that INTS1, INTS2, INTS3, INTS4, INTS5, INTS7, INTS8, INTS9, INTS11 (CPSF3L), INTS12, INTS13 (C12orf11), and INTS14 (C15orf44) were highly expressed in tumor tissues compared to adjacent normal tissues. Conversely, INTS10 expression levels were reduced, and INTS6 showed no statistical significance, as shown in Supplementary Fig. 1. To verify these results, we cross-checked the data obtained with the data collected in the GEPIA2 system. At an enhanced *P* value of 0.05, we found that only INTS1, INTS3, INSTS4, INTS7, and INST8 were highly expressed in tumor tissues compared to normal tissues, as shown in Fig. 1A.

Furthermore, metastasis often results in a much worse prognosis for cancer patients. Therefore, we analyzed the differential expression of INTS1, INTS3, INTS4, INTS7, and INTS8 in normal, further tumor, and metastatic HCC tissues using the TNM plotter. The results showed that the expression of INTS1, INTS7, and INTS8 transcripts in both HCC tumors and HCC metastatic tumors was significantly higher than that in normal tissues (Fig. 1B). Unfortunately, INTS4 was not in the database.

We then examined the expression of proteins associated with INTS1, INTS3, INTS4, INTS7, and INTS8 in HCC through the Clinical Proteomic Tumor Analysis Consortium (CPTAC) and Human Protein Atlas (HPA) databases. CPTAC database analysis indicated that the protein levels of INTS1, INTS3, INTS4, INTS7, and INTS8 were higher in HCC tissues than in normal liver tissues (*P* < 0.001) (Supplementary Fig. 2A). Using the HPA database, we observed upregulated expression of INTS1 and INTS8 proteins in HCC tissue compared to normal liver tissue, while INTS7 protein expression was not detected in the HPA database (Supplementary Fig. 2B). Epigenetic regulation through DNA methylation is known to modulate gene expression status. As promoter methylation plays a key role in HCC progression, we analyzed the expression and promoter methylation levels of the INTS family using the UALCAN tool from the TCGA dataset. Our analysis revealed that the promoter of INTS6 and INTS9 were hypermethylated in HCC tissues, while the promoter of INTS1, INTS2, INTS5, INTS7, and INTS14 were hypomethylated in HCC tissues compared to normal tissues as shown in Supplementary Fig. 3.

### INTS1, INTS3, INTS4, INTS7, and INTS8 mRNA expression in HCC cells

**Fig. 2:**
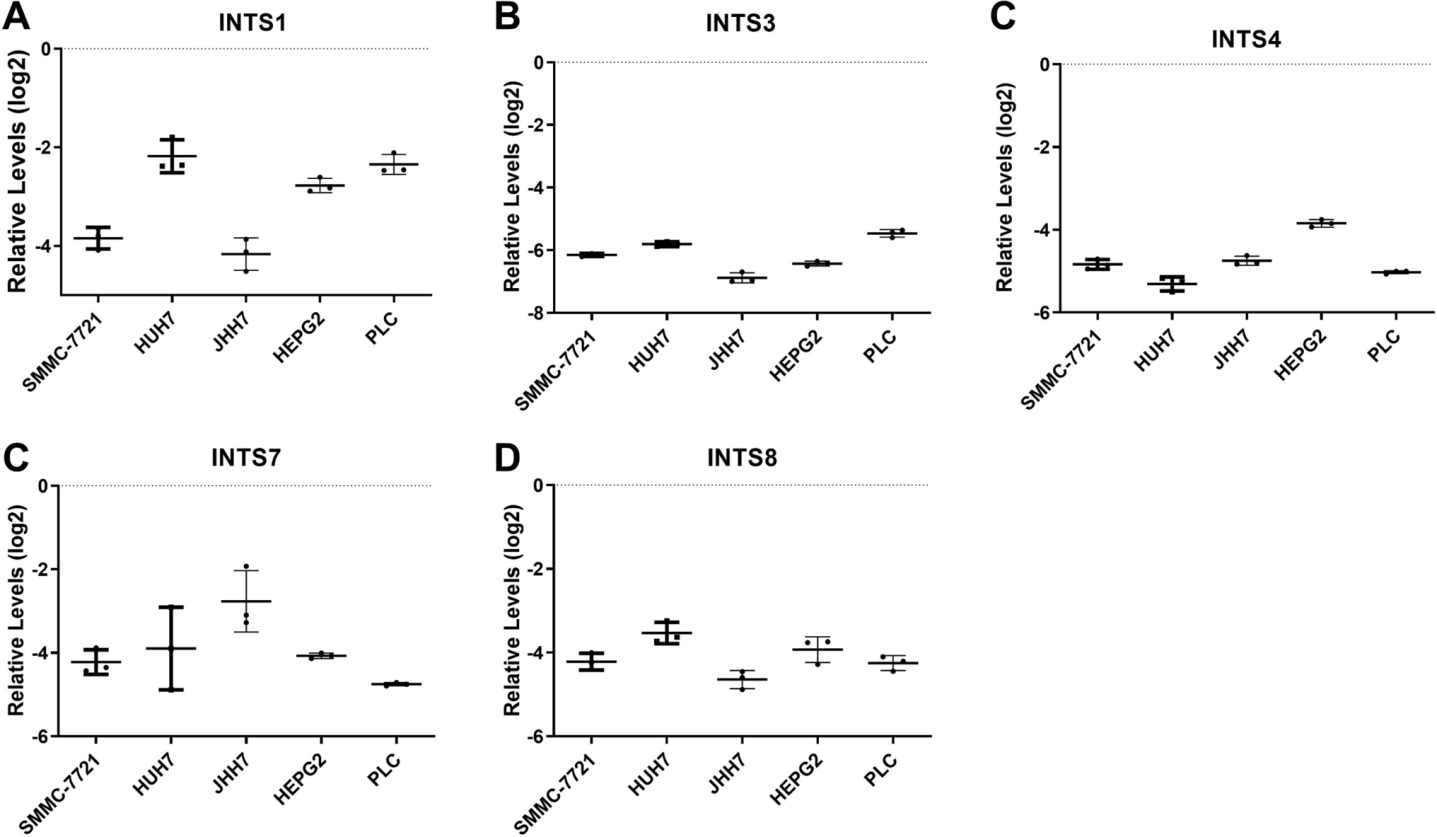
Relative expression levels of INTS1, INTS3, INST4, INTS7 and INST8 detected by qRT-PCR. **A–D** The expression levels of INTS1, INTS3, INST4, INTS7 and INST8 in 5 HCC cell lines (SMMC-7721, HUH7, JHH7, HepG2 and PLC/PRF/5) detected by qRT-PCR (n = 3). The median level of expression is represented in each group by a solid line. The expression values are normalized to GAPDH expression levels and presented on the Y-axis in a log 2 scale

We examined the mRNA expression levels of INTS1, INTS3, INTS4, INTS7, and INTS8 in SMMC-7721, HUH7, JHH7, HepG2, and PLC/PRF/5 HCC cells. The results showed that INTS1, INST4, INTS7, and INST8 expression was expressed in high abundance in HCC cells (Fig. 2). Accordingly, INTS1, INSTS4, INTS7, and INST8 were selected for further analysis.

### Correlation of the expression of INTS family members with clinicopathologic features of HCC patients

**Fig. 3:**
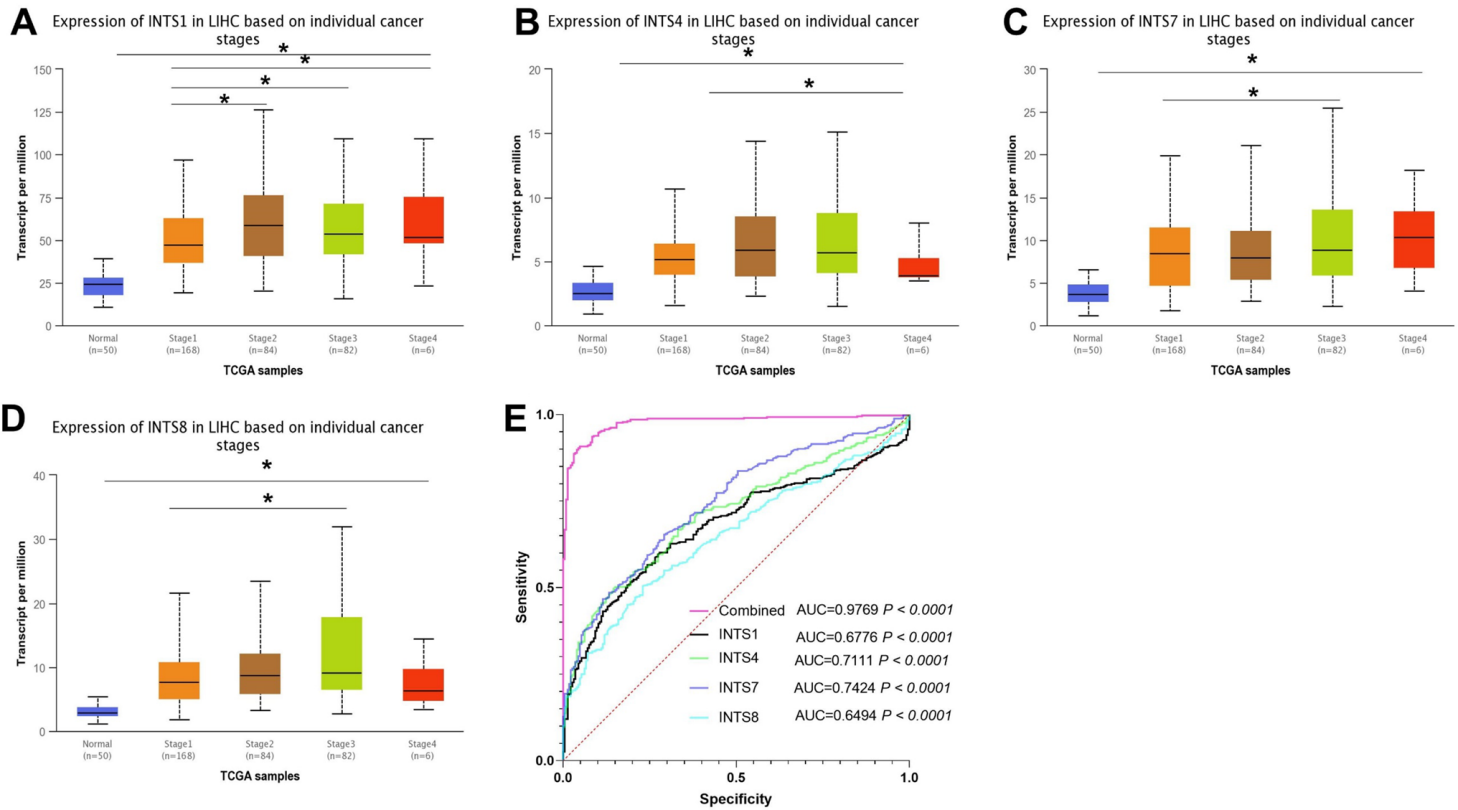
Relationship between the expression levels of NTS1, INTS4, INTS7 and INTS8 and the pathological stage of HCC patients from UALCAN database

Subsequently, we investigated whether the expression levels of INTS1, INTS4, INTS7, and INTS8 correlated with patient tumor staging. Correlation analysis of TCGA data using the UALCAN database revealed that INTS1, INTS4, INTS7, and INTS8 expression was correlated with the pathological stages of HCC patients (Fig. 3). The expression of INTS1, INTS4, INTS7, and INTS8 increased with tumor progression, with all mentioned genes reflecting statistical differences in at least two groups of liver cancer tumor grades comparison. These results suggest the plausibility of using these genes as clinicopathological parameters to assist in determining tumor grades in HCC. ROC curve analysis was utilized to ascertain the diagnostic value of INTS1 in distinguishing HCC tissues (n = 423) from non-HCC tissues (n = 226). The area under the curve (AUC) of the TCGA HCC cohort for INTS1, INTS4, INTS7, and INTS8 were AUC = 0.6776, *P* < 0.0001; AUC = 0.7111, *P* < 0.0001; AUC = 0.7424, *P* < 0.0001; AUC = 0.6494, *P* < 0.0001, respectively. The combination of INTS1, INTS4, INTS7, and INTS8 had a very high accuracy rate, with an AUC of 0.9769, *P* < 0.0001. Consequently, the combination of INTS1, INTS4, INTS7, and INTS8 emerged as a reliable diagnostic marker in HCC.

### The prognostic value of the INTS family in HCC patients

**Fig. 4:**
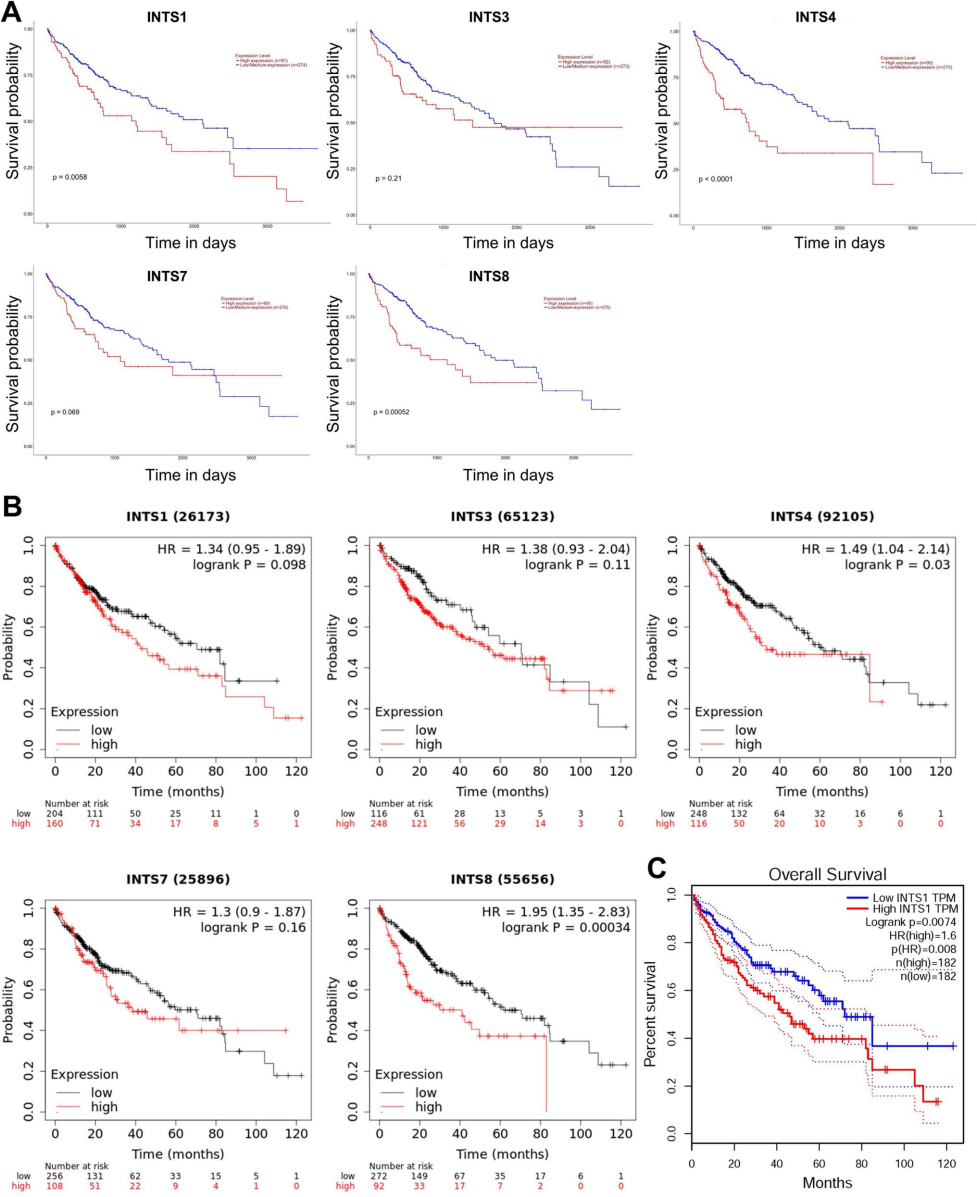
Prognostic value of INTS1, INTS3, INTS4, INTS7 and INTS8 in patients with HCC from UALCAN, KM-plotter and GEPIA databases

Accordingly, we conducted a comprehensive analysis to determine the prognostic value of INTSs in HCC patients using the UALCAN, KM-plotter, and GEPIA databases. The results indicated that high expression levels of INTS1, INTS4, and INTS8 were strongly associated with poor survival outcomes, as demonstrated by UALCAN (Fig. 4A), high expression levels of INTS4 and INTS8 were strongly associated with a poor survival situation from KM-plotter (Fig. 4B), and high expression level of INTS1 were strongly associated with a poor survival situation from GEPIA (Fig. 4C), respectively. These findings suggest that high expression levels of INTS1, INTS4, and INTS8 serve as potential biomarkers for predicting the survival of HCC patients and are strongly associated with HCC patient prognosis.

### The genetic alteration and mutation of four-INTSs in HCC

**Fig. 5:**
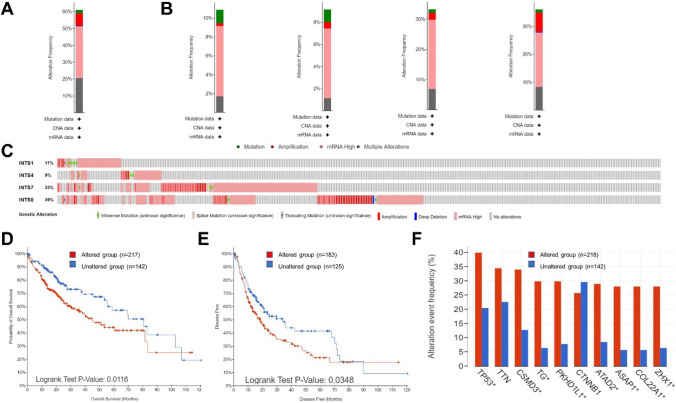
The genetic alteration and mutation of four-INTSs in HCC using cBioPortal database. **A** Total alteration frequency of the four-INTSs in HCC. **B, C** Individual alteration frequency of four-INTSs in HCC. **D, E** The prognostic analysis of the genetic alteration of the four-INTSs in HCC, including overall (*P* = 0.0116) and disease-free survival (*P* = 0.0348). F Genes with highest frequency between altered group (n = 218) and unaltered group (n = 142). Recent studies have demonstrated the significant involvement of the INTSs in transcription regulation, nucleic acid metabolism, and various human diseases, including neurodevelopmental disorders, postmenopausal osteoporosis, and malignancies such as HCC

We analyzed the genetic alteration and mutation of four-INTSs in HCC through the cBioPortal database. Our analysis indicated that genetic alteration in four-INTSs accounted for more than 61% in HCC patients (Fig. 5A). The genetic alterations included mutation, amplification, mRNA low, mRNA high, and multiple alterations, with an average of 10% accounted for by each gene (Fig. 5B, C). The progression of genetic alterations in the four-INTSs indicated that the altered group had poorer Overall Survival (OS) (*P* = 0.0116) and Disease-Free Survival (DFS) (*P* = 0.0348) than the unaltered group (Fig. 5D, E and Supplementary Table S2). Additionally, we observed that the altered group had a higher frequency of typical HCC gene mutations such as TP53, CSMD3, TG, PKHD1L1, ATAD2, ASAP1, COL22A1, and ZHX1 (Fig. 5F).

Furthermore, we analyzed the difference in sample-level enrichments between the unaltered and altered groups, with 950 genes obtained in the altered group using *P* < 0.05 and log ratio > 2 as screening conditions. The top 20 differently enriched genes that emerged from our analysis included ESRP1, CALB1, PDP1, NBN, RALYL, and DECR1, among others. These genes have been implicated in tumor onset and progression (Supplementary Table S3) [29–34].

### Immune cell infiltration of four-INTSs in patients with HCC

**Fig. 6:**
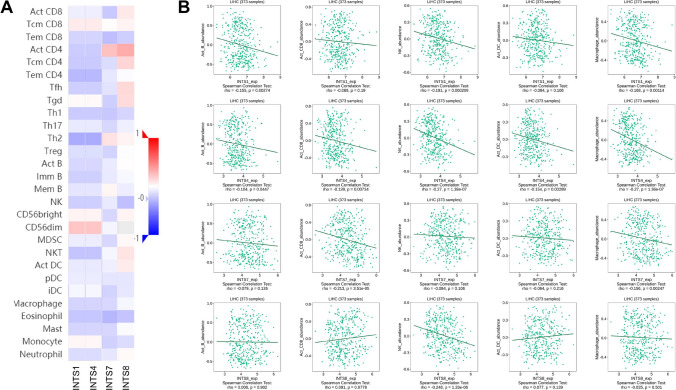
Association of four-INTSs expression with immune infiltration level in HCC from TISIDB. **A** Heat map of immune infiltrate correlation for four-INTSs. **B** Scatter plot of the correlation between the expression level of four-INTSs and immune cell infiltration, including Act_B cells, Act_CD8 + T cells, NK cells, Act DC, macrophages (Spearman correlation, n = 373). Act, activated; NK, natural killer cells; DC, dendritic cells

We investigated the relationship between the expression of four INTSs (INTS1, 4, 7, 8) and immune infiltration levels in HCC using the TIMER database. Our analysis revealed that INTS1, INTS4, INTS7, and INTS8 showed a positive association with B cells, macrophages, dendritic cells, and CD4 + T cells. Additionally, INTS4, INTS7, and INTS8 exhibited a positive correlation with neutrophils and CD8 + T cells (Supplementary Fig. 4). Inflammation and immune cell infiltration play critical roles in the clinical prognosis of cancer patients. Tumor-infiltrating immune cells in the tumor microenvironment can be divided into tumor-promoting immune cells and anti-tumor immune cells. During tumor progression, the high expression of specific oncoproteins in tumor cells can recruit immunosuppressive lymphocytes, such as MDSCs and regulatory T cells (Tregs), and enhance their immunosuppressive function. Furthermore, it can render tumor-killing lymphocytes, such as CD8 + T cells and NK cells, anergic, thus creating a suitable ecological environment for tumor growth. Therefore, we observed a contradiction where genes with negative prognostic implications were found to have a positive association with immune infiltration, specifically CD8 + T cells, which typically indicate a favorable clinical outcome. We hypothesized that these findings might be attributed to the activation status and subgroups of immune cells. To gain deeper insights, we conducted a comprehensive immune infiltration analysis of the four-INTSs in HCC using the TISIDB databases, focusing on different functional subgroups. Our analysis revealed that, on the whole, the expression of the four-INTSs displayed a negative correlation with various immune infiltrations, primarily activated B cell, activated CD8 + T cell, and macrophage infiltration (Fig. 6A, B). These findings suggest that the expression of the four-INTSs may be associated with a suppressive tumor immune microenvironment in HCC.

### Gene correlation expression and mutations with the INTS family’s expression in HCC

We utilized various databases such as UALCAN, GEPIA2, HPA, TNMplot, and KM-plotter to analyze the expression and mutations associated with the INTS family of genes in HCC. Our analysis indicated that INTS1, INTS4, INTS7, and INTS8 were highly expressed in HCC tissues, with their expressions closely correlated with tumor grade and poor prognosis of HCC patients. Additionally, PCR results also showed that INTS1, INTS4, INTS7, and INTS8 were highly expressed in HCC cells, with INTS1 being the most highly expressed. To further investigate the expression and mutations associated with the INTS family of genes, we focused our study on four genes, namely INTS1, INTS4, INTS7, and INTS8. Using UALCAN, we studied the relationship between the promoter methylations of these genes and the TP53 mutation status in HCC. Our analysis showed that only INTS1's promoter methylations coincide with the TP53 mutation status and have statistical significance, as depicted in Supplementary Fig. 5. These findings implicate that INTS1 may nest in the P53 pathway affecting the progression of HCC.

### Analysis of four-INTSs co-expressed genes and their functional enrichment in HCC

**Fig. 7:**
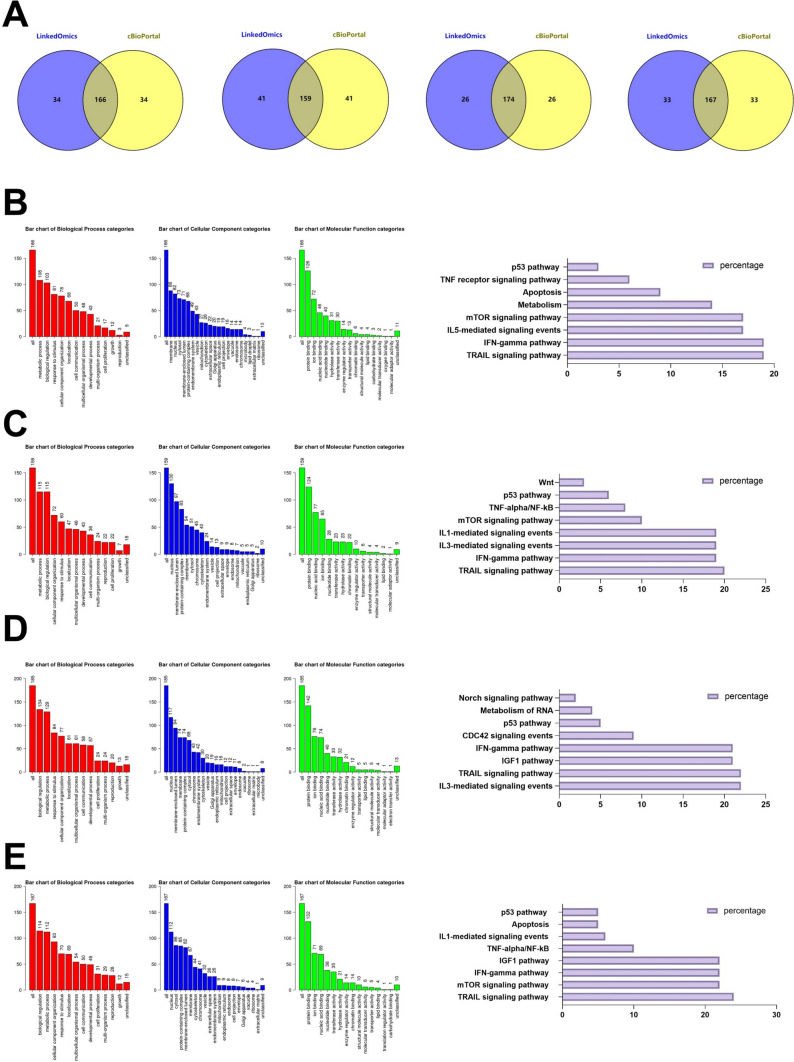
Comprehensive bioinformatics analysis of four-INTSs and individual co-expressed genes in HCC. **A** Intersection of genes positively co-expressed in the top 200 of the LinkedOmics and the cBioPortal database (Spearman correlation). **B–E** The GO enrichment analysis and KEGG pathway prediction of four-INTSs were performed. BP, biological processes; CC, cellular components; MF, molecular function

After analyzing the expression and mutations associated with the INTS family of genes, we narrowed our study down to four genes, namely INTS1, INTS4, INTS7, and INTS8, which were differentially expressed between the tumor and normal cell lines, had potential as prognostic markers and were associated with various tumor-grade stages. Using the LinkedOmics database and the cBioPortal database, we screened the top 200 positively co-expressed genes and obtained the intersected genes (Fig. 7A). We predicted the functions and pathways of four-INTSs by performing the GO and KEGG enrichment analysis. The GO enrichment analysis includes three points, namely BP, CC, and MF. For INTS1, BP terms indicated metabolic process, biological process, cell communication, cell proliferation, and growth. CC terms were implicated in the membrane, nucleus, and cytosol. MF terms were involved in protein binding, ion binding, and nucleotide binding. The KEGG pathways showed that INTS1 was related to the TRAIL, IFN-gamma, IL5-mediated signaling events, Apoptosis, and p53 pathway (Fig. 7B). For INTS4, BP terms were implicated in metabolic process, biological regulation, cell proliferation, and growth. CC terms were involved in the nucleus, membrane-enclosed lumen, and protein-containing complex. MF terms contained protein binding, nucleic acid binding, and ion binding. The KEGG pathways indicated that INTS4 was related to TRAIL, IFN-gamma, IL3-mediated signaling events, mTOR, and p53 pathway (Fig. 7C). For INTS7, BP terms were implicated in biological regulation, metabolic process, response to stimulus, cell proliferation, and growth. CC terms were involved in the nucleus, membrane-enclosed lumen, and membrane. MF terms contained protein binding, ion binding, and nucleic acid binding. The KEGG pathways indicated that INTS7 was related to the IL3-mediated signaling events, TRAIL, IGF1, IFN-gamma, CDC42, and p53 pathway (Fig. 7D). For INTS8, BP terms were implicated in biological regulation, metabolic process, and cellular component organization. CC terms were involved in the nucleus, cytosol, and protein-containing complex. MF terms contained protein binding, ion binding, and nucleic acid binding. The KEGG pathways indicated that INTS8 was related to TRAIL, mTOR, IFN-gamma, IGF1, TNF-alpha/NF-kB, IL1-mediated signaling events, Apoptosis, and p53 pathway (Fig. 7E).

Based on our findings, we can observe that the co-expressed genes of INTS1, 4, 7, and 8 are involved in various processes related to cancer development. This suggests that the focused genes INTS1, 4, 7, and 8 may have a significant role in the progression of HCC. Furthermore, our analysis shows that these related genes are closely associated with pathways related to cancer progression, highlighting the crucial importance of the INTS gene family in HCC research and the potential for novel progress.

### Phenotypic evaluation of INTS1, INTS4, INTS7 and INTS8 with HCC

Carcinogenesis is a multistage process initiated by disturbed and uncontrolled proliferation of cells. The growth of these cells is associated with the actions of proliferative proteins such as proliferating cell nuclear antigen (PCNA) and Ki-67. Therefore, we used the TNMplot database to examine the correlation between the expression of PNCA, KI67, and INTS1, INTS4, INTS7, and INTS8. Our analysis revealed that the expression of PNCA increases parallel to the elevated expression of the studied genes. Additionally, we observed that the elevation of INTS1, INTS4, INTS7, and INTS8 expression occurs in parallel with an increase in KI67, as shown in Supplementary Fig. 6A. Furthermore, we analyzed the DepMap database and identified that the knockout of INTS1, INTS4, INTS7, and INTS8 using CRISPR/Cas9 significantly suppressed the growth of HCC cell lines (Supplementary Fig. 6B).

### Knockdown of INTS1 inhibits cell proliferation in HCC

**Fig. 8:**
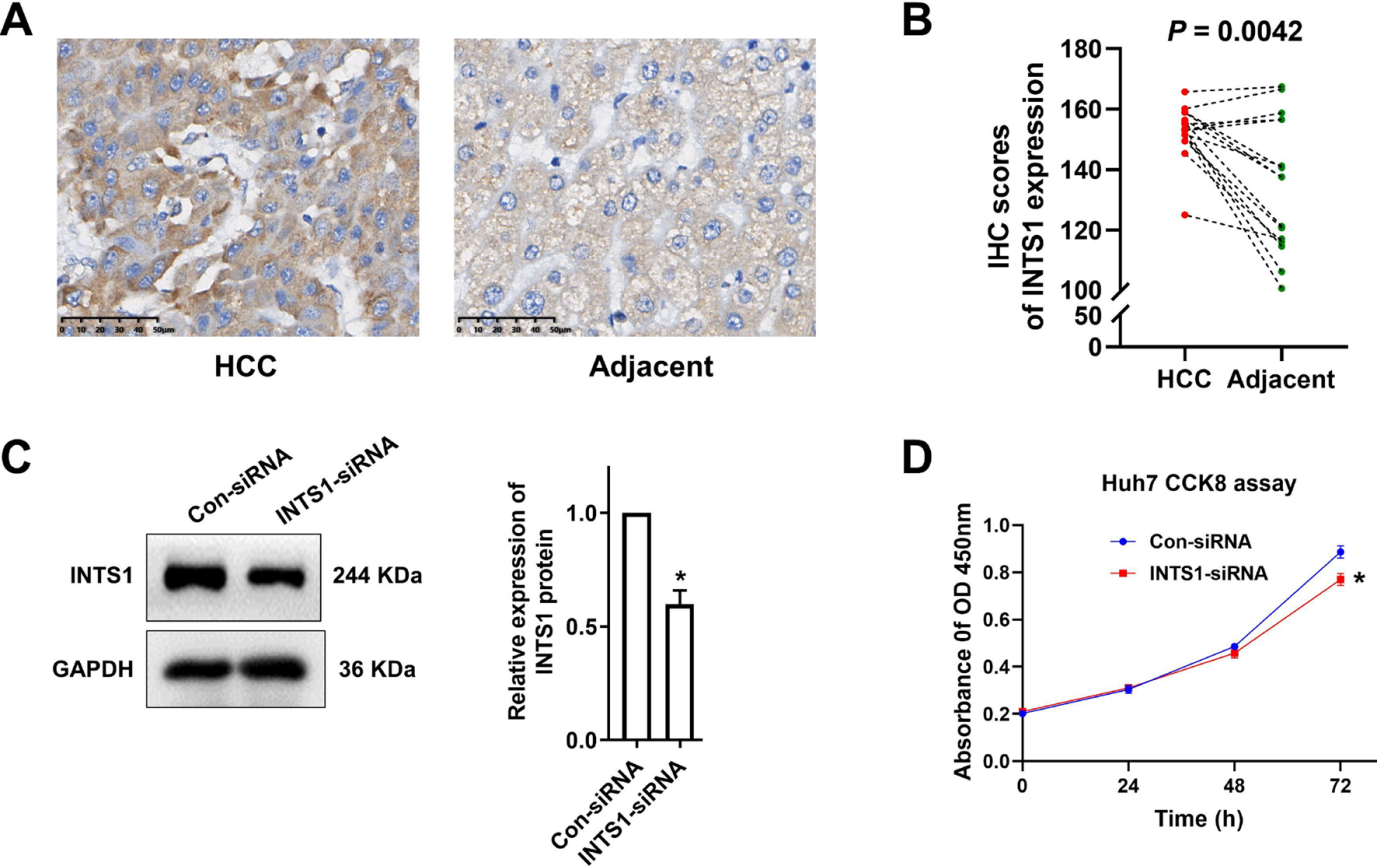
Expression of INTS1 protein in HCC tissue and knockdown of INTS1 inhibits cell proliferation in HCC. **A** The scan of a classic pair of HCC tissues and adjacent tissues after INTS1 immunohistochemistry staining (IHC). **B** INTS1 protein expression in 16 pairs of HCC tissues and adjacent tissues, as determined by IHC. **C** Western blot analysis shows the knockdown of INTS1 in HUH7 cells by the use of specific siRNA for INTS1. The bottom panel represents the relative quantification of INTS1 for three replicates. The data are presented as mean ± SD. *P < 0.05. **D** Knockdown of INTS1 inhibited cell proliferation of Huh7 cells, as evaluated by the CCK-8 assay. * *P* < 0.05

Our previous bioinformatics analysis and qRT-PCR results have indicated the potential crucial role of INTS1 in the development of HCC. As a result, we decided to conduct further investigations on INTS1 based on these findings. By employing immunohistochemistry (IHC), we examined the protein expression of INTS1 in 16 HCC tissues and their corresponding adjacent tissues. Our analysis revealed significantly higher levels of INTS1 protein in cancerous tissues compared to adjacent non-cancerous tissues (Fig. 8A and B). Additionally, HUH7 cells exhibited a higher mRNA expression level of INTS1 (Fig. 2). To explore the biological functions of INTS1, we utilized siRNA to knockdown INTS1 in HUH7 cells. The knockdown efficiency was validated through Western blot analysis (Fig. 8C). Subsequently, we conducted a CCK8 assay, which demonstrated that the suppression of INTS1 led to a significant decrease in the proliferation of HUH7 cells (Fig. 8D).

## Discussion

HCC, one of the most prevalent cancers globally, lacks effective treatment options, highlighting the urgent need for new therapeutic targets. The INTSs are protein complex found in eukaryotic cells, and they play a crucial role in gene expression regulation. The INTSs are responsible for processing and integrating small nuclear RNAs (snRNAs) transcribed by RNA polymerase II. The INTSs consists of approximately 14 subunits (INTS1-14) that work together to perform its functions. The primary function of the INTSs is to cleave and polyadenylate the 3' end of the snRNAs, which are important for RNA splicing. This processing step ensures the stability and functionality of the snRNAs. Additionally, the INTSs also help in associating the cleaved snRNAs with the snRNP particles, which are necessary for pre-mRNA splicing [35, 36]. Furthermore, recent studies have suggested that the INTSs might play a broader role in transcription regulation [37]. It has been implicated in the modulation of gene expression, including the regulation of protein-coding genes [38]. Recent studies have demonstrated the significant involvement of the INTSs in transcription regulation, nucleic acid metabolism, and various human diseases, including neurodevelopmental disorders, postmenopausal osteoporosis, and malignancies such as HCC.

The homozygous INTS1 mutation (c.5351C > A, p (S1784*)) is associated with rare recessive human neurodevelopmental syndromes, which lead to abnormal brain development and malformations of cortical development [39]. Homozygous deletion of INTS1 results in embryonic developmental arrest at an early blastocyst or morula stage [40]. A GWAS screening identified the INTS2 mutation (p.G906K) in gastric cancer peritoneal carcinomatosis [41]. Overexpression of INTS3 has been observed in HCC [42]. It is involved in DNA damage repair and G2/M checkpoint activation [43]. INTS4 is responsible for maintaining the integrity of Cajal bodies and histone locus bodies [44]. INTS5 is closely associated with postmenopausal osteoporosis [45]. Knockdown of INTS5 affects the development of the hematopoietic system by regulating the BMP signaling pathway in zebrafish [46]. INTS6 exhibits significantly down-regulated expression levels in non-small cell lung cancer and prostate cancer compared to normal tissues [47]. In esophageal squamous cell carcinoma, somatic mutations have been found in conserved functional domains, and tumor cell SNPs are also associated [48]. INTS6 induces the formation of adipose cells [49] and participates in DNA damage repair and G2/M checkpoint activation [50]. In C. elegans, the encoded protein localizes to the mitochondrial inner membrane and is involved in the remodeling of mitochondrial crest. Its deficiency leads to mitochondrial morphological abnormalities and apoptosis. INTS6 is also essential for nematode oogenesis, late embryogenesis, and larval growth [51]. Additionally, the level of DICE1 mRNA is higher in bovine ovarian dominant follicular granulosa cells compared to those undergoing apoptosis, indicating the anti-apoptotic effect of bovine DICE1 protein [52]. INTS7 plays a role in repairing DNA damage [53]. INTS8 has two mutations, c.893 A > G (p.D298G) and c.2917_2925del (p.E972_L974del), which are associated with rare recessive human neurodevelopmental syndromes [54]. The expression level of INTS8 in gastric cancer and paracancerous tissues suggests that it could be used as an early diagnostic molecule for gastric cancer [55]. Mutation p.W548L is associated with peripheral T-cell lymphoma based on GWAS screening` [56]. INTS8 is overexpressed and accelerates the epithelial-to-mesenchymal transition in HCC [57]. INTS9, when regulated through the BMP signaling pathway in zebrafish, affects the development of the hematopoietic system [58]. INTS10 might be a nicotine-dependent genetic susceptibility gene [59]. Additionally, it was identified as a germline susceptibility locus in childhood precursor B-cell acute lymphoblastic leukemia INTS11 in zebrafish knockdowns resulted in hematopoietic dysfunction and inducing the formation of adipose cells. INTS12 was found to be significantly correlated with susceptibility to chronic obstructive pulmonary disease. INTS13 plays a role in the assembly of dynein and nuclear membranes. INTS14 expression is increased in SV40-immortalized cells, lung cancer cells, and non-small cell lung cancer tissues.

However, the precise biological role of INTSs in HCC remains unclear. In this study, we discovered that INTS1, INTS4, INTS7, and INTS8 exhibited high expression levels in tumor tissues compared to normal tissues in both the UALCAN and GEPIA2 systems (*P* < 0.05). Moreover, the expression of INTS1, INTS7, and INTS8 transcripts was significantly higher in both primary HCC tumors and HCC metastatic tumors compared to normal tissues, with the expression level in HCC metastatic tumors being significantly higher than in primary HCC tumors. The protein expression of INTS1 and INTS8 was also upregulated in HCC tissue when compared to normal liver tissue, as supported by the CPTAC and HPA database. Analysis of the promoter methylation levels of the INTS gene family in HCC tissues revealed hypermethylation of the INTS6 and INTS9 promoters, while the promoters of INTS1, INTS2, INTS5, INTS7, and INTS14 exhibited hypomethylation compared to normal tissues. These findings were confirmed through qRT-PCR analysis of five HCC cell lines, which displayed upregulation of INTS1, INTS4, INTS7, and INTS8 compared to a normal liver cell line. Considering these results, we selected INTS1, INTS4, INTS7, and INTS8 for further investigation.

Additionally, previous research has conducted a bioinformatic study focusing on the INTS family’s character on the overall and survival of the HCC patient, and showed that the INTS has the potential of performing as an independent signature in the realm of clinical practice of cancer management [60]. Our analysis suggests that elevated expression levels of INTS1, INTS4, and INTS8 could potentially serve as biomarkers for predicting the survival of HCC patients and are strongly correlated with prognosis. Genetic alterations in these four INTS genes were observed in over 61% of HCC patients. Additionally, we discovered a negative association between the expression of these INTS genes and various immune infiltrations, indicating a suppressive tumor immune microenvironment in HCC. In relation to TP53 mutation status, we investigated the promoter methylations of INTS1, INTS4, INTS7, and INTS8, and only INTS1's promoter methylation coincided with TP53 mutation status, suggesting its involvement in the P53 pathway, which affects HCC progression (Supplementary Fig. 5). Furthermore, we observed that the co-expressed genes of INTS1, INTS4, INTS7, and INTS8 were involved in diverse processes associated with cancer development, underscoring their significant role in HCC progression. These related genes exhibited close associations with pathways related to cancer progression, which highlights the importance of the INTS gene family in HCC research.

We investigated the relationship between the expression of PNCA, KI67, INTS1, INTS4, INTS7, and INTS8 in HCC using the TNMplot database. The results showed that INTS1, INTS4, INTS7, and INTS8 expression was positively correlated with KI67 and PCNA expression in HCC. Moreover, data from the DepMap database suggests that knockout of INTS1, INTS4, INTS7, and INTS8 using CRISPR/Cas9 significantly inhibits the growth of HCC cells. We chose to further investigate INTS1 as it was found to be highly expressed in HCC tissues compared to non-cancerous tissue. Subsequent knockdown of INTS1 in HUH7 cells resulted in decreased cell proliferation. These results imply that INTS1, alongside INTS4, INTS7, and INTS8, play crucial roles in HCC development and could potentially serve as biomarkers and therapeutic targets for the disease.

Despite the thoroughness of our study and the use of multiple public databases and experimental techniques, there are limitations that should be acknowledged. Firstly, data from public databases may contain biases or inconsistencies that could impact the accuracy and reliability of our findings. Additionally, sample sizes in certain experimental analyses, such as immunohistochemistry and RT-qPCR, were limited, which may affect result generalizability. Furthermore, while our study identified significant associations between INTS expression and various clinicopathological characteristics in HCC, the mechanisms underlying these correlations remain unclear. Future studies should further investigate the potential therapeutic implications of targeting INTS1, INTS4, INTS7, and INTS8 in HCC, as these genes play a crucial role in disease progression and prognosis. Understanding the molecular mechanisms driving INTS upregulation in HCC and their interactions with other signaling pathways is also essential. By exploring the crosstalk between INTS genes and cancer-related pathways, valuable insights into their oncogenic properties and potential therapeutic targets can be gained. Investigating the regulatory relationships among miRNAs, lncRNAs, and the INTS family in HCC could enhance our understanding of hepatocarcinogenesis and provide new avenues for precision medicine in treatment. Additionally, validating INTS1 as a biomarker for HCC diagnosis and prognosis is crucial given its correlation with tumor stages and patient outcomes. Confirming INTS1 as a reliable biomarker could improve early detection and management of HCC in clinical practice.

## Conclusions

In conclusion, our study highlights the significance of INTS family genes in HCC pathogenesis and their potential as diagnostic, prognostic, and therapeutic targets. Further research in this area has the potential to advance our understanding of HCC biology and contribute to the development of precision medicine approaches for treating this deadly disease.

### Supplementary Information

Additional file 1.

## Data Availability

The datebases we used including TCGA (https://portal.gdc.cancer.gov/), cBioPortal (http://www.cbioportal.org/), GEPIA2 (http://gepia2.cancer-pku.cn/), Kaplan–Meier Plotter (http://kmplot.com), LinkedOmics (http://linkedomics.org/login.php), TISIDB (http://cis.hku.hk/TISIDB/index.php). and the UCSC Xena browser (https:// xenab rowser. net/ datap ages/) were all public databases, in which the date in our study could be downloaded and analysed.

## Abbreviations

HCC: Hepatocellular carcinoma
INTS: Integrator complex subunit
GO: Gene ontology
KEGG: Kyoto encyclopedia of genes and genomes
HPA: Human protein atlas
FPKM-UQ: Fragments per kilobase of transcript per million mapped reads for the upper quartile
LIHC: Liver hepatocellular carcinoma
GTEx: Genotype-tissue expression project
IHC: Immunohistochemistry
CPTAC: Clinical proteomic tumor analysis consortium
DFS: Disease-free survival
OS: Overall survival
snRNA: Small nuclear RNA
