# Supplementary material for: Comprehensive bioinformatics analysis of integrator complex subunits: expression patterns, immune infiltration, and prognostic signature, validated through experimental approaches in hepatocellular carcinoma
Source: Discov Oncol. 2024 Jun 26;15:246. doi: 10.1007/s12672-024-01118-6 (PMC11208364; doi:10.1007/s12672-024-01118-6)
Supplement: Supplementary file 1 — Additional file 1. [file 12672_2024_1118_MOESM1_ESM.docx]

Supplementary Material

Comprehensive Bioinformatics Analysis of Integrator Complex Subunits: Expression Patterns, Immune Infiltration, and Prognostic Signature, Validated through Experimental Approaches in Hepatocellular Carcinoma

**Yifei Xu^1^** **· Wenlian Liao^1^ · Ting Wang^1^** **· Liwei Zhang^2^ · Hui Zhang^1^**

^1^ Institute of Interdisciplinary Integrative Medicine Research, Shanghai University of Traditional Chinese Medicine, Shanghai 201203, China

^2^ Department of Anesthesiology, Xiang'an Hospital of Xiamen University, School of Medicine, Xiamen University, Xiamen, Fujian 361101, China

Yifei Xu and Wenlian Liao have contributed equally to this work.

🖂 Hui Zhang

zhanghuiman@126.com

🖂 Liwei Zhang

14159078@qq.com

# Supplementary Figures and Tables

## Supplementary Figures


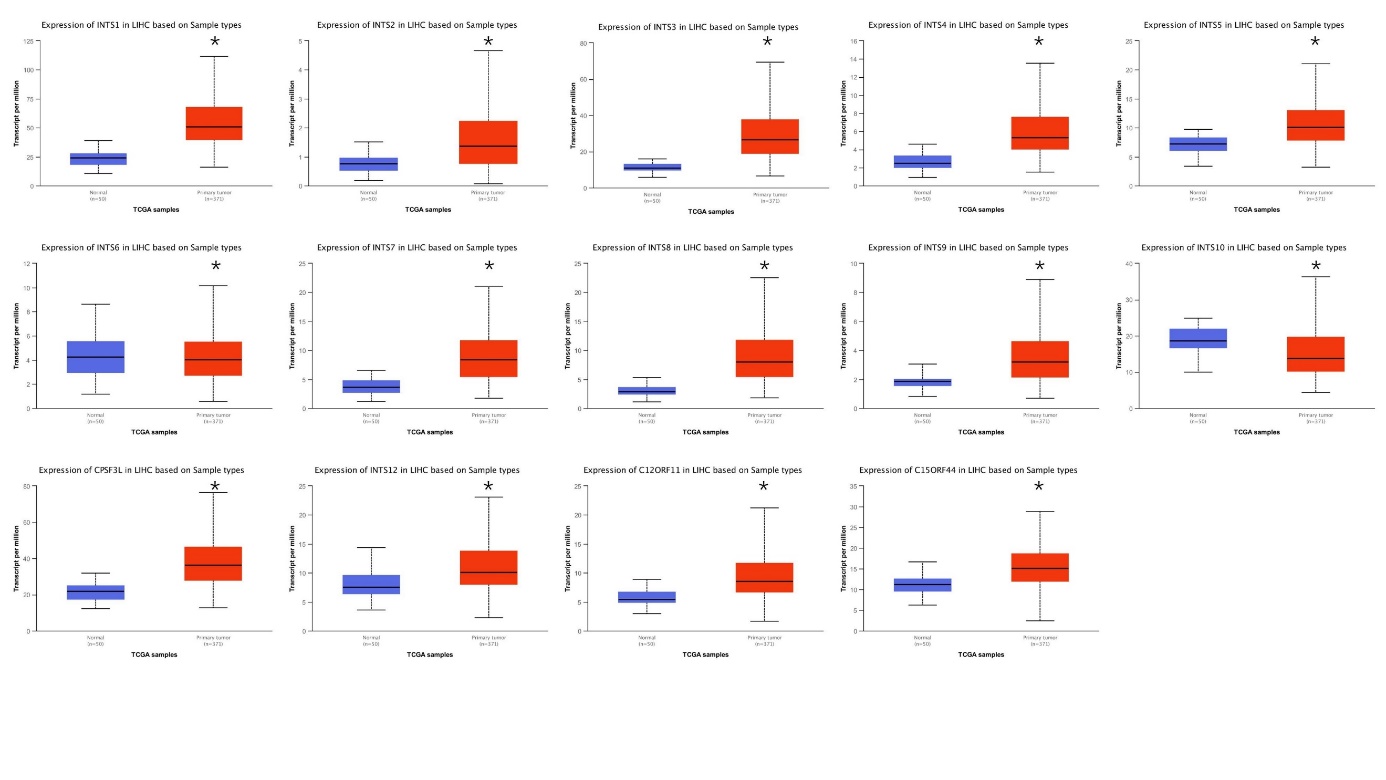


**Supplementary** **Fig. 1** The mRNA expression levels of INTS family (INTS1-14) in HCC tissues (n = 371) versus normal liver tissues (n = 50) using UALCAN database. **P* < 0.05.


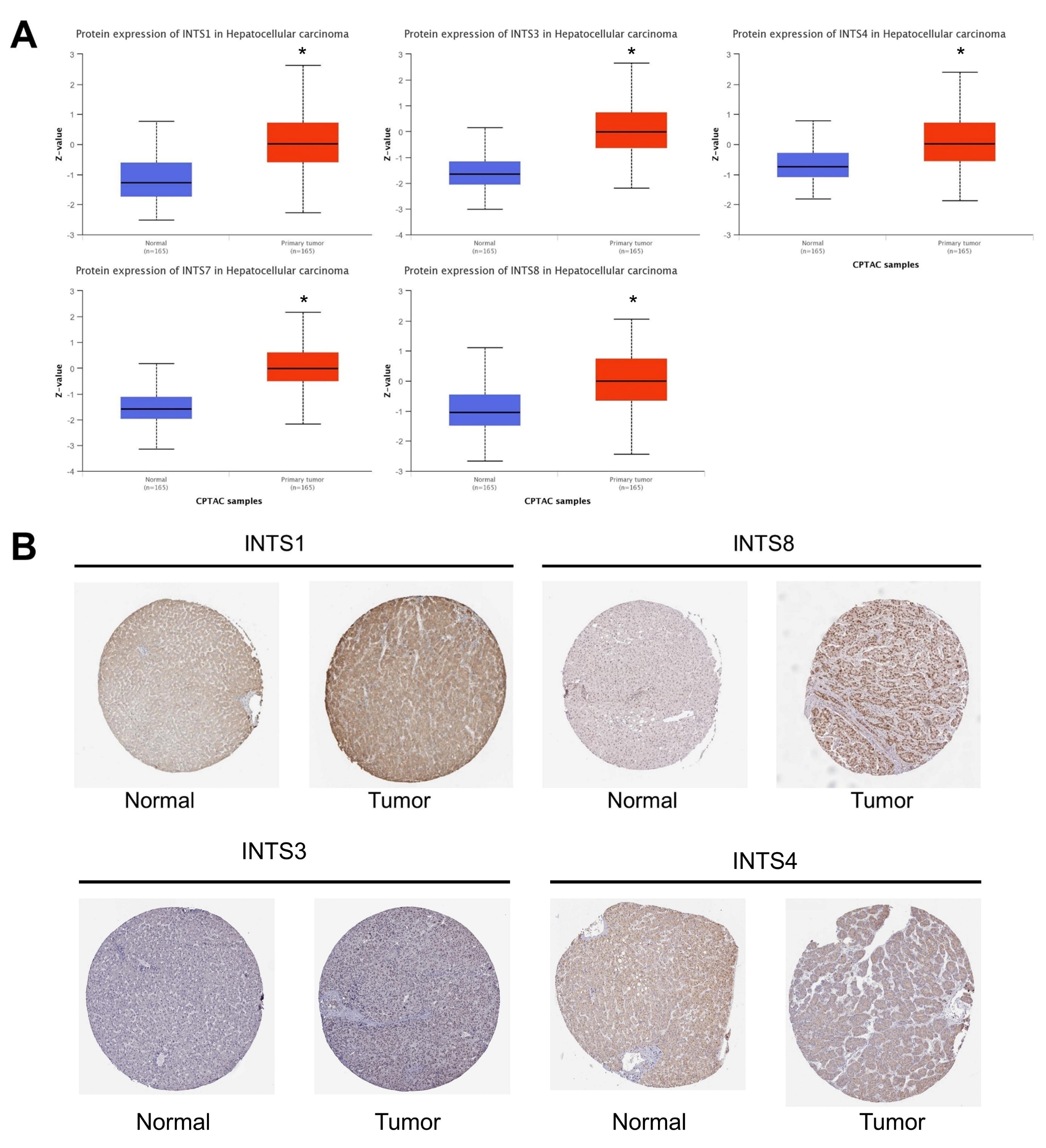


**Supplementary Fig. 2** The protein expression levels of INTS1, INTS3, INTS4, INTS7 and INTS8 in HCC tissues and normal liver tissues from CPTAC (**A**) and Human Protein Atlas (**B**) databases. INTS1 antibody: HPA021658. INTS3 antibody: HPA074391. INTS4 antibody: HPA042378. INTS8 antibody: HPA057299. **P* < 0.05.


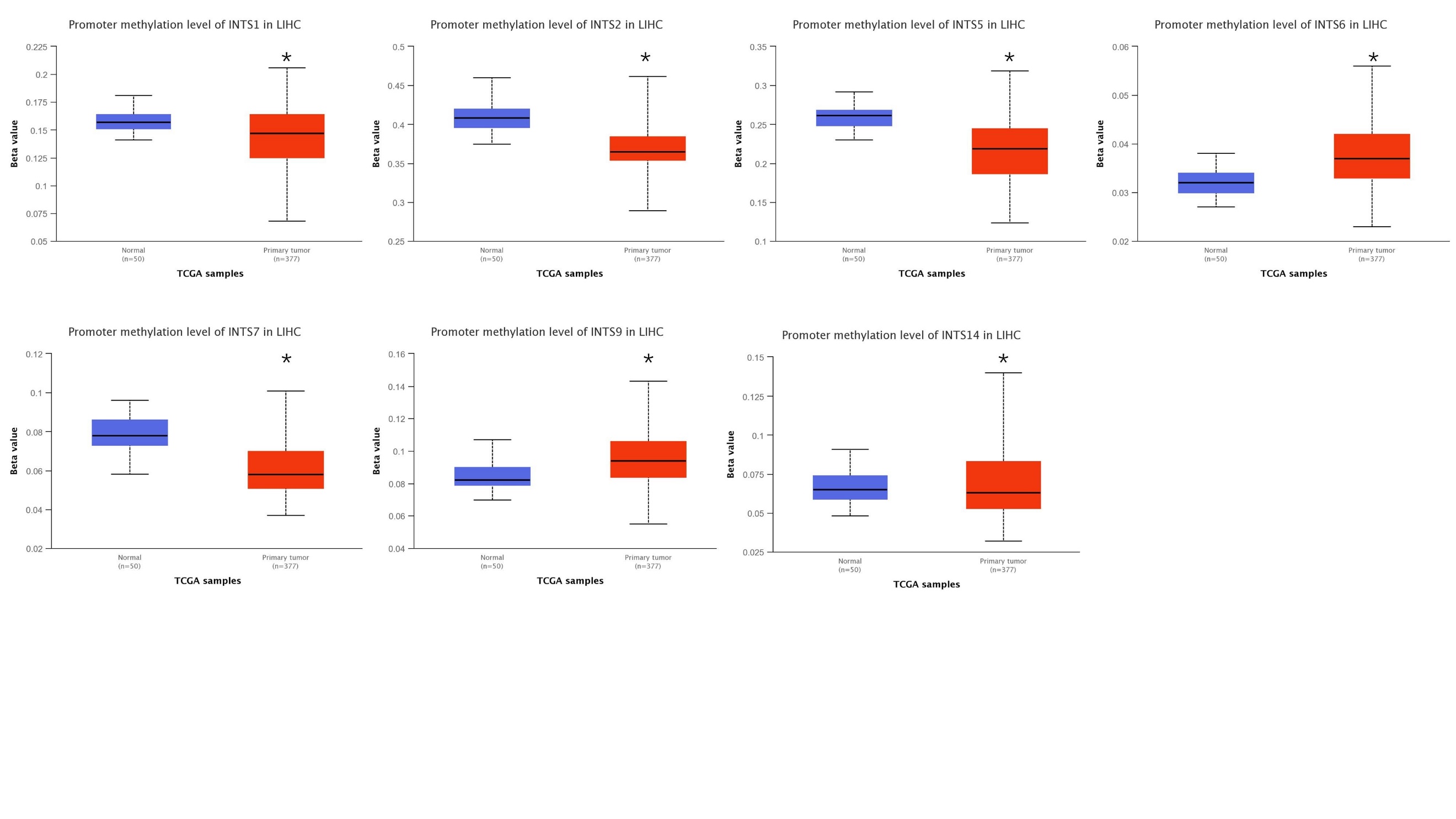


**Supplementary Fig. 3** The mRNA expression of INTSs and promoter methylation in HCC. Box plot and *P* value were produced using UALCAN. **P* < 0.05.


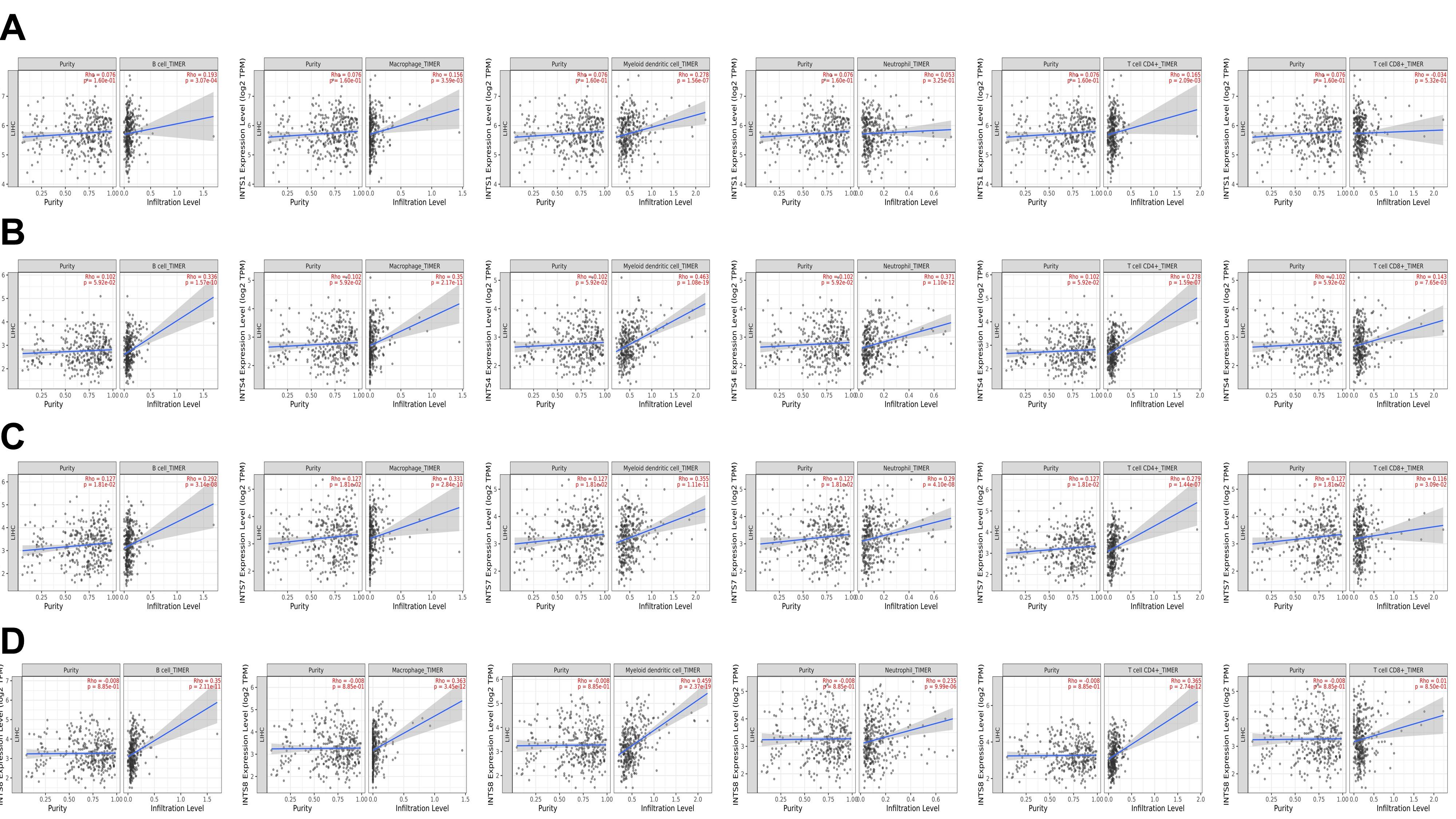


**Supplementary Fig. 4** Association of four-INTSs expression with immune infiltration level in HCC from TIMER database.


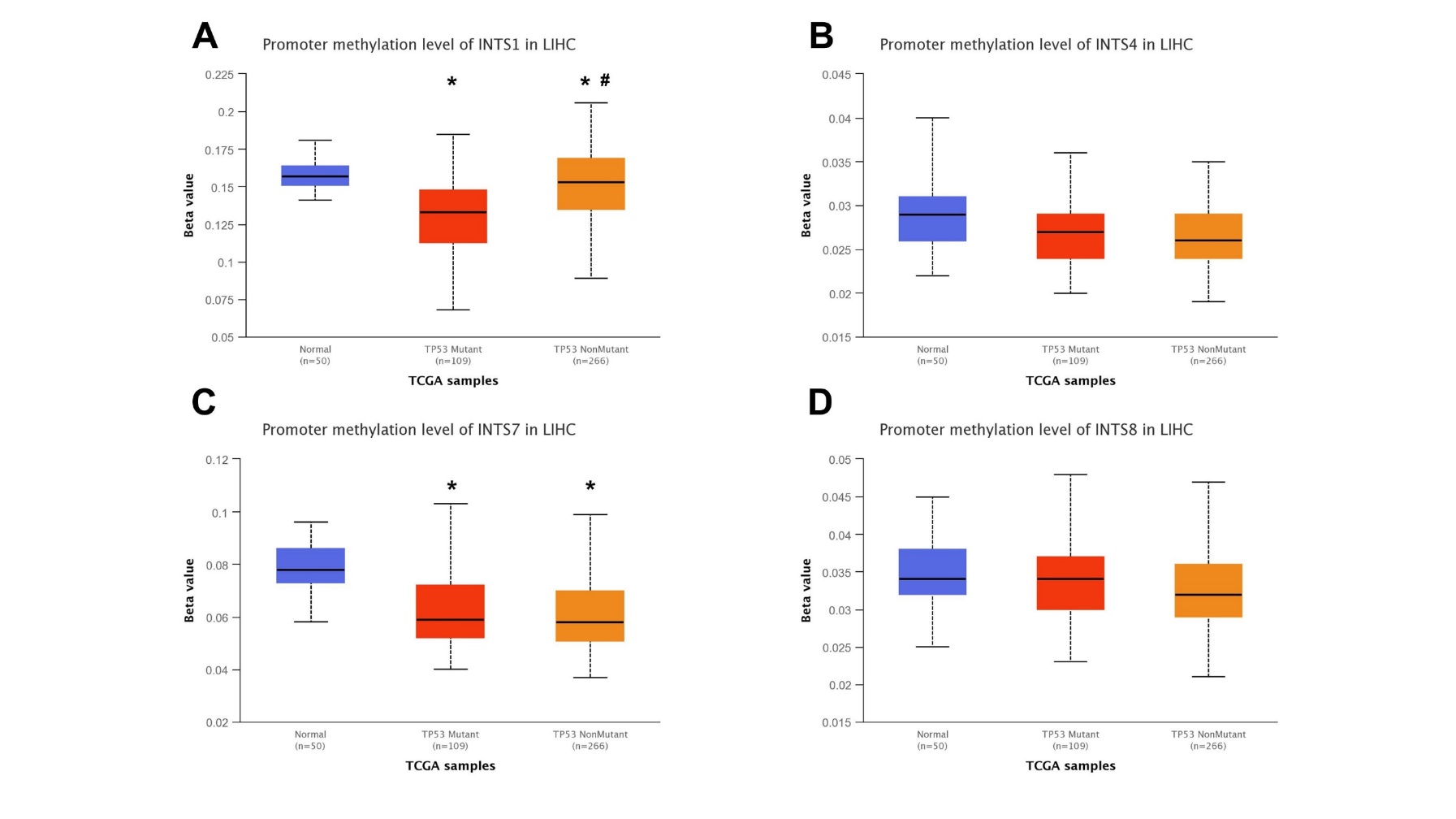


**Supplementary Fig. 5** Correlations of INTS1, 4, 7 and 8 promoter methylation levels with TP53 mutation status.


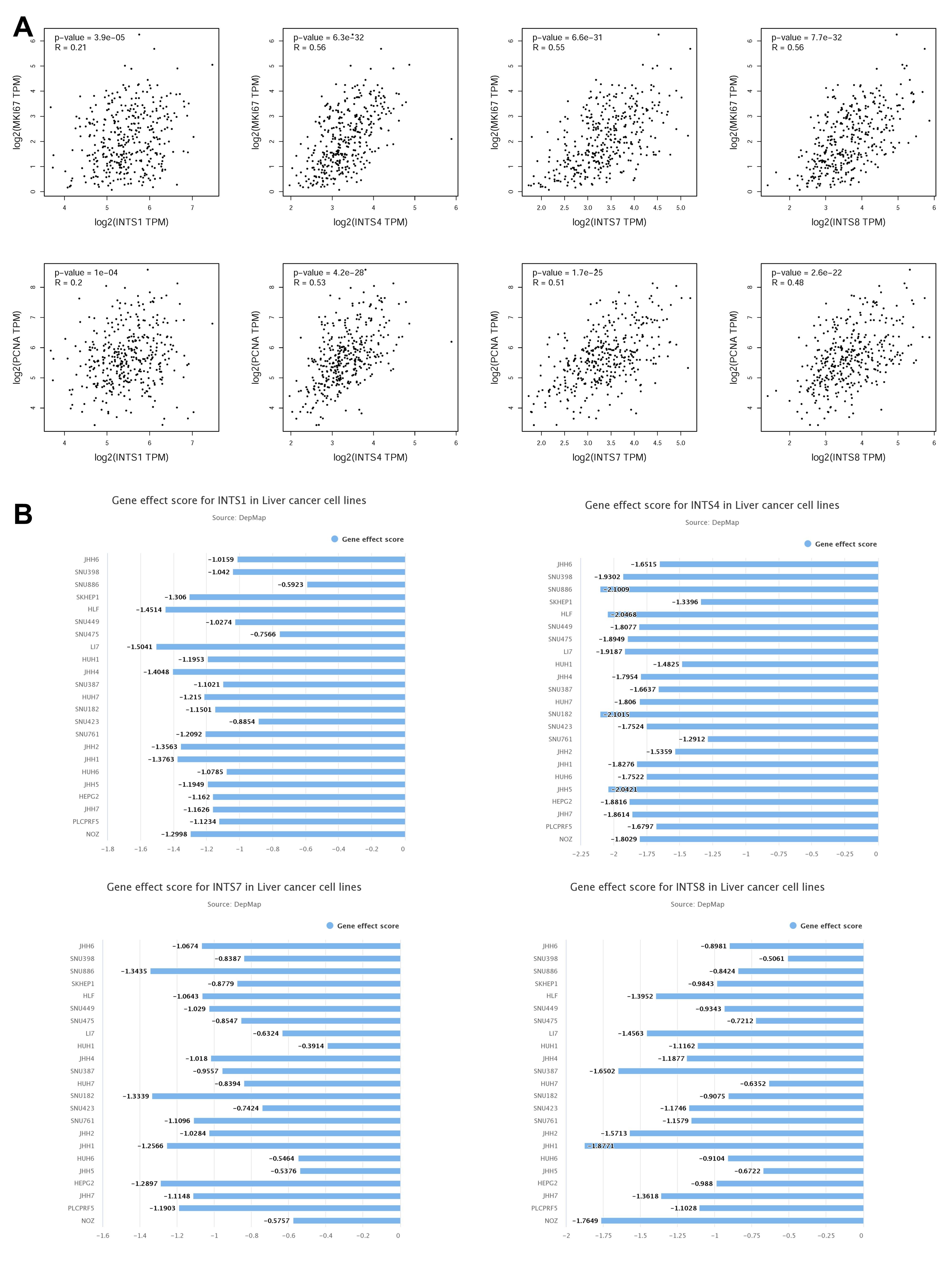


**Supplementary Fig. 6** Phenotypic evaluation of INTS1, INTS4, INTS7 and INTS8 with HCC. **A** The correlation expression between PNCA, KI67 and INTS1, INTS4, INTS7 and INTS8 was examined using TNMplot. **B** Knockout of INTS1, INTS4, INTS7 and INTS8 using CRISPR/Cas9 could significantly suppress the growth of HCC cell lines by analyzing the DepMap database.

## Supplementary Tables

**Supplementary Table S1 | Primer sequences for qRT-PCR**.

| **Gene name** | **Sequence of primer (5' to 3')** |
| --- | --- |
| INTS1-FP | ACCTGCTGATGTCCGTCTG |
| INTS1-RP | GCAGTGCGGTATAGAGGACC |
| INTS2-FP | AACTCATCCTTCGCCTTCTTTC |
| INTS2-RP | GTTTATGCCTAAGCTGCTGTTCT |
| INTS3-FP | TCAATGCGTATGTGTGCAAAGG |
| INTS3-RP | AAGTCCCGGTAACACTTCTGG |
| INTS4-FP | TCTGTGTAAAGCTACCTCCCC |
| INTS4-RP | GAATCCTGACTACTCCCTCTACG |
| INTS7-FP | TGATTCAAGGTTTAGCGACACC |
| INTS7-RP | TGTAAAAGCTGACGAGCACTG |
| INTS8-FP | TGGCTATTGTCAAGCATGTGAT |
| INTS8-RP | TTACCTCCTGATAGTTGCCAGA |
| GAPDH-FP | GGAGCGAGATCCCTCCAAAAT |
| GAPDH-RP | GGCTGTTGTCATACTTCTCATGG |

qRT-PCR: quantitative reverse transcription-polymerase chain reaction; FP: forward primer; RP: reverse primer.

**Supplementary Table 2. Prognostic analysis of four-INTSs between altered group and unaltered group from cBioportal in HCC**

| **Survival Type** | | **Number of Patients** | **Altered group** | **Unaltered group** | **Median months survival in Altered group (95% CI)** | **Median months survival in Unaltered group (95% CI)** | ***p*-Value** | ***q*-Value** |
| --- | --- | --- | --- | --- | --- | --- | --- | --- |
| Overall | 359 | | 217 | 142 | 45.07 (33.02 - NA) | 80.68 (55.65 - NA) | 0.0116 | 0.0231 |
| Disease Free | 308 | | 183 | 125 | 18.33 (13.07 - 25.30) | 33.90 (20.89 - 71.06) | 0.0348 | 0.0348 |

**Supplementary Table 3. The top 20 genes between TNTS family altered group and unaltered group in HCC.**

| **Gene** | **Cytoband** | **Altered group** | **Unaltered group** | **Log2 Ratio** | **p-Value** | **q-Value** | **Enriched in** |
| --- | --- | --- | --- | --- | --- | --- | --- |
| ESRP1 | 8q22.1 | 56 (25.69%) | 0 (0.00%) | >10 | 2.48E-14 | 4.70E-10 | Altered group |
| CALB1 | 8q21.3 | 54 (24.77%) | 0 (0.00%) | >10 | 8.66E-14 | 4.70E-10 | Altered group |
| DPY19L4 | 8q22.1 | 54 (24.77%) | 0 (0.00%) | >10 | 8.66E-14 | 4.70E-10 | Altered group |
| GEM | 8q22.1 | 54 (24.77%) | 0 (0.00%) | >10 | 8.66E-14 | 4.70E-10 | Altered group |
| CIBAR1 | 8q22.1 | 53 (24.31%) | 0 (0.00%) | >10 | 1.61E-13 | 5.83E-10 | Altered group |
| PDP1 | 8q22.1 | 53 (24.31%) | 0 (0.00%) | >10 | 1.61E-13 | 5.83E-10 | Altered group |
| CIBAR1-DT | 8q22.1 | 52 (23.85%) | 0 (0.00%) | >10 | 2.99E-13 | 6.49E-10 | Altered group |
| LINC02906 | 8q22.1 | 52 (23.85%) | 0 (0.00%) | >10 | 2.99E-13 | 6.49E-10 | Altered group |
| RNA5SP274 | 8q22.1 | 52 (23.85%) | 0 (0.00%) | >10 | 2.99E-13 | 6.49E-10 | Altered group |
| TRIQK | 8q22.1 | 52 (23.85%) | 0 (0.00%) | >10 | 2.99E-13 | 6.49E-10 | Altered group |
| NBN | 8q21.3 | 51 (23.39%) | 0 (0.00%) | >10 | 5.53E-13 | 8.58E-10 | Altered group |
| PIP4P2 | 8q21.3 | 51 (23.39%) | 0 (0.00%) | >10 | 5.53E-13 | 8.58E-10 | Altered group |
| RALYL | 8q21.2 | 51 (23.39%) | 0 (0.00%) | >10 | 5.53E-13 | 8.58E-10 | Altered group |
| TMEM64 | 8q21.3 | 51 (23.39%) | 0 (0.00%) | >10 | 5.53E-13 | 8.58E-10 | Altered group |
| DECR1 | 8q21.3 | 50 (22.94%) | 0 (0.00%) | >10 | 1.02E-12 | 1.01E-09 | Altered group |
| LINC00534 | 8q21.3 | 50 (22.94%) | 0 (0.00%) | >10 | 1.02E-12 | 1.01E-09 | Altered group |
| OSGIN2 | 8q21.3 | 50 (22.94%) | 0 (0.00%) | >10 | 1.02E-12 | 1.01E-09 | Altered group |
| RIPK2 | 8q21.3 | 50 (22.94%) | 0 (0.00%) | >10 | 1.02E-12 | 1.01E-09 | Altered group |
| RN7SKP231 | 8q21.3 | 50 (22.94%) | 0 (0.00%) | >10 | 1.02E-12 | 1.01E-09 | Altered group |
| PSKH2 | 8q21.3 | 49 (22.48%) | 0 (0.00%) | >10 | 1.88E-12 | 1.44E-09 | Altered group |
